# Neurophysiological evidence for rapid processing of verbal and gestural information in understanding communicative actions

**DOI:** 10.1038/s41598-019-52158-w

**Published:** 2019-11-08

**Authors:** Rosario Tomasello, Cora Kim, Felix R. Dreyer, Luigi Grisoni, Friedemann Pulvermüller

**Affiliations:** 1 Brain Language Laboratory, Department of Philosophy and Humanities, WE4 Freie Universität Berlin, Habelschwerdter Allee 45, 14195 Berlin, Germany; 2 Berlin School of Mind and Brain, Humboldt Universität zu Berlin, Luisenstraße 56, 10117 Berlin, Germany; 3 Cluster of Excellence ‘Matters of Activity. Image Space Material’, Humboldt Universität zu Berlin, Unter den Linden 6, 10099 Berlin, Germany; 4 Einstein Center for Neurosciences, Charitéplatz 1, 10117 Berlin, Germany

**Keywords:** Neuroscience, Language

## Abstract

During everyday social interaction, gestures are a fundamental part of human communication. The communicative pragmatic role of hand gestures and their interaction with spoken language has been documented at the earliest stage of language development, in which two types of indexical gestures are most prominent: the *pointing* gesture for directing attention to objects and the *give-me* gesture for making requests. Here we study, in adult human participants, the neurophysiological signatures of gestural-linguistic acts of communicating the pragmatic intentions of naming and requesting by simultaneously presenting written words and gestures. Already at ~150 ms, brain responses diverged between naming and request actions expressed by word-gesture combination, whereas the same gestures presented in isolation elicited their earliest neurophysiological dissociations significantly later (at ~210 ms). There was an early enhancement of request-evoked brain activity as compared with naming, which was due to sources in the frontocentral cortex, consistent with access to action knowledge in request understanding. In addition, an enhanced N400-like response indicated late semantic integration of gesture-language interaction. The present study demonstrates that word-gesture combinations used to express communicative pragmatic intentions speed up the brain correlates of comprehension processes – compared with gesture-only understanding – thereby calling into question current serial linguistic models viewing pragmatic function decoding at the end of a language comprehension cascade. Instead, information about the social-interactive role of communicative acts is processed instantaneously.

## Introduction

An essential aspect of human communication is the use of indexical hand gestures and their combination with speech to communicate a variety of intentions in social interactions. Much of previous empirical work has strongly supported the idea that gestures influence language understanding and that verbal language and gesture systems interact in an integrative process^1,2^. Event-related potential (ERP) studies have documented early and late brain indicators of semantic processing during language-gesture comprehension (for a review see Özyürek *et al*.^3^). In particular, these studies have reported an N400 effect in semantic violation paradigms, when gestures were incongruent with preceding speech segments^4–10^, and in disambiguation paradigms, when gestures where disambiguating upcoming speech content^11,12^. Some of this research suggested that the semantic-pragmatic integration of gestures into ongoing speech is (to some degree) automatic^9^. However, most of the aforementioned neurocognitive studies focused on a subset of gestures, the so-called iconic gestures, which are characterized by an intrinsic relationship between form and meaning^1,13,14^. For instance, if a person produces an iconic hand gesture outside speech context - e.g. a round hand shape - its meaning may be quite vague, as it could be meant to refer to an apple, a wheel or a ball. However, if it is used with the sentence ‘I will eat an apple’, its meaning is focused by context, but its use is partly redundant. Such context dependent vagueness or redundancy is less prominent for a different intensely investigated class (e.g., Gunter *et al*.,^4^; Proverbio *et al*.^10^), that is, symbolic or ‘emblematic’ gestures whose semantic meaning is arbitrarily related to their form; these are typically used autonomously, outside speech context, or in replacement of speech (e.g., thumb up)^1,14–16^. However, these types of gesture are semantically similar to verbal signs, most of which are also symbols. In the current study, we focused on hand gestures that are typically used together with verbal signs and, as language-gesture combination, serve a main function of transmitting pragmatic communicative intentions. These are called *indexical gestures* and, if they are used together with words to refer to specific objects, *deictic gestures*^1,14,17,18^ (see also Kelly *et al*.^19^). The pointing gesture is the most representative of this category, which is typically used in ostensive definition (‘this is a cone’) and an equally important item is the whole hand pointing or ‘give-me’ gesture typically used to express requests (‘give me this/the cone’)^17,20–24^. Despite the fact that the neural signatures of language and iconic and symbolic gestures have been studied by a body of previous research, little is known about the neurobiology of indexical and deictic gestures and their interplay with verbal language use in transmitting information about specific communicative intentions in adults’ social interaction.

To close this gap, we investigated the neurophysiological basis of two types of gestures: the *pointing* gesture for directing attention to an object and the *give-me* gesture for requesting a desired object^17,20–24^. In children, these two gestures are associated with the communicative intentions of naming and requesting^20,25–28^. The separate and specific contributions gestures and words make when used in combination can best be described in terms of linguistic pragmatic theories that define communicative functions in terms of their illocutionary force and their propositional content^29,30^. When communication is carried by the simultaneous use of words and indexical gestures, the pointing or give-me gesture is frequently informative about communicative function (or speech act) type or illocutionary force^29,30^, indexing an assertive or directive role respectively. In contrast, the verbal materials (‘spoon’ or ‘milk’) make it clear what the communication is about, i.e., what the (propositional) content is^31–33^ (e.g., give-me gesture + ‘spoon’, for requesting a spoon, pointing + ‘milk’ for naming the content of a cup).

In recent years, the communicative pragmatic function of language has become a key topic in neurocognitive research^34–39^. Brain correlates of communicative functions carried by linguistic utterances have been investigated, revealing, for example, different brain areas activated when subjects understand the same utterances either as request or naming actions^40^. A key observation from this earlier work was that the greater action affordance of request actions—which were expected to be followed by a handing-over of the requested object —is reflected in increased activation of the motor system as compared with a naming context. This result raises the question whether distinct brain indicators specific to speech act types arise in a similar way when the same communicative function is carried by combinations of basic hand gestures and words rather than by verbal-linguistic material alone.

Cognitive and neurobiological theories of communication and language provide a further and most important theory-driven reason to look closely at the brain mechanisms of language-gesture interplay: Linguists and cognitive scientists have long debated at which exact point in time in the understanding process information about communicative function first comes into play. Most current psycholinguistic models of language comprehension^41–43^ (see also models of language production^44,45^) assume a cascaded processing timeline, according to which the (onset of the) analysis of phonological form precedes lexical access and morpho-syntactic analysis, which are followed by semantic comprehension, and, only at the end of the processing cascade, pragmatic understanding. Crucially, the delays between the different levels is not assumed to be in the range of milliseconds but rather in the range of 100 s of milliseconds, thus explaining a delay of ca. 400 ms from presentation of written language to the emergence of semantic brain indexes such as the N400 (see, for example, Friederici 2011^42^). Models differ as to whether this cascade is strict and serial^41,42^, or rather of a more flexible nature^43^, but the final position of pragmatic in the processing cascade is shared by different approaches. As an alternative to seriality, other models proposed parallel or near-simultaneous processing of the different subtypes of psycholinguistic information within 200 ms in comprehension^46–51^ and also in production^52,53^. It is therefore of utmost importance to work toward deciding between these cascaded vs parallel theories based on experimental results. In this context, a crucial question is whether pragmatic access to information about communicative function and content is a slow or rapid process.

Previous neurophysiological studies revealed brain dynamics in the millisecond range and indicated that a physiological difference between verbal speech acts performed by using the same linguistic utterances is reflected by early brain responses, within the first 200 ms after presentation of the linguistic stimulus^54,55^. This early pragmatic dissociation result speaks in favour of parallel models. However, a caveat of these earlier studies lies in the fact that speech act function was determined by a preceding context, so that a ‘context sentence’ (for example ‘What are these called?’ for naming or ‘What can I get you?’ for requesting) had made it clear before critical sentence onset, which communicative function this linguistic utterance would carry. As, in these previous experiments, the observed very early brain responses to the critical stimuli could have been influenced (primed) by the pre-existing predictive knowledge about speech act function, one may still ask whether communicative function processing indeed represents an early step in the understanding process under conditions where communicative function is not predictable from previous context and the communicative act itself first reveals the information about its illocutionary role. Here, the simultaneous use of gesture and language comes in handy, as information about communicative content and function can be provided exactly at the same time, through words and gestures, respectively. Such simultaneous availability is critical for addressing the theoretical key question about the time course of communicative function understanding in the mind and brain.

The present study seeks to explore the time course and cortical origin of neurophysiological activity underlying, and distinguishing between, the comprehension of two fundamental communicative acts, naming and requesting, expressed by the interplay between gestural indexes and linguistic symbols. We expect a ready and quick differentiation between request and naming acts expressed through gesture-word combination, supporting parallel instead of serial models of language processing. Furthermore, we predict stronger neural sources to requests than to naming in the sensorimotor regions relevant for action processing, a finding which would converge with previous neurocognitive studies on communicative action understanding in verbal only paradigms^34,35,40,55^. Gestures presented outside linguistic context will be used to control for the processing of information intrinsic to the gestures.

## Results

### Behavioural results

Performance on catch trails, in which the participants had to respond to the presented communicative acts appropriately (see Methods section ‘*stimuli and procedure*’ for more details), was highly accurate (92% SE = 0.18), demonstrating that the participants were understanding and paying attention to the items displayed on the screen.

### GFP analysis

**Figure 1:**
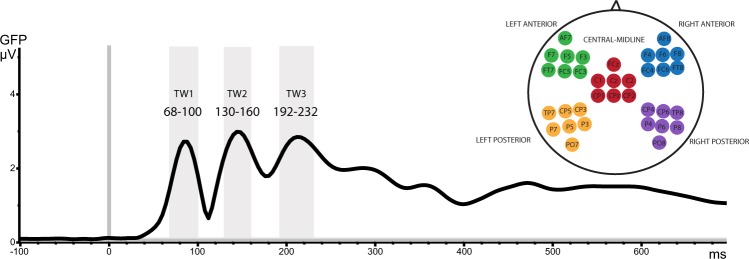
Selection of time windows for statistical analysis. Global field power (GFP) computed across all EEG electrodes, averaged across all subjects and conditions. The highlighted time windows of interest were defined around the identified GFP peaks using the Full Width at Half Maximum (FWHM). On the right-hand side, the electrode selection for the topographical analysis is shown divided into: left anterior (LA), right anterior (RA), central midline (CM), left posterior (LP) and right posterior (RP) subsets.

Peaks revealed by the GFP waveform computed over all conditions and electrodes were found at 80 ms, 143 ms and 210 ms. Calculated as the FWHM around these peaks, the resultant time windows were (1) 68–100 ms, (2) 130–160 ms and (3) 192–232 ms (see Fig. 1). An additional pre-defined time window was placed at 300–500 ms (N400), as a small peak with long latency was revealed at ca. 400 ms. This late pre-defined N400-window was analysed separately from the early data-driven time windows.

#### Early effects, gestures-only

**Figure 2:**
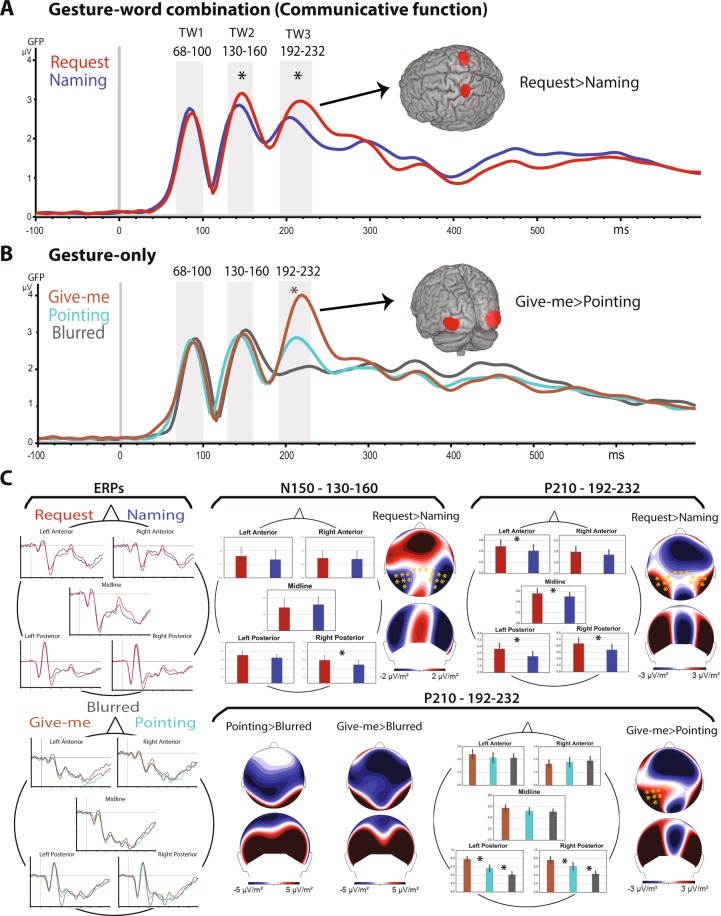
Main results. (**A**) Global field power waveforms of the gesture-word conditions request (in red) and naming (in blue). The depicted brain illustrates the results of the whole brain source analysis. (**B**) (grand-average) GFP waveforms of the Gesture alone conditions give-me (in brown), pointing (in light blue), and blurred (in grey). The depicted brain illustrates the results of the whole brain source analysis. (**C**) ERPs on the left-hand side and bar graphs on the right hand-side illustrating the significant results of the topographical analysis in each time window; error bars show standard errors, asterisks indicate the significant difference between conditions (Bonferroni planned comparison tests). Topographical maps (current source density, CSD) show differences in event-related potential distributions between conditions for each of the time windows and the orange asterisks indicate the electrodes that survived the cluster-based permutation test.

The three data-driven time windows were used for the statistical analysis of the gesture-only conditions. Firstly, the gesture-only conditions (blurred, pointing, give-me gestures) were statistically investigated in a 2-way ANOVA (Time window × Gesture type) calculated across the three time windows, which revealed a highly significant interaction (F(4, 96) = 8.47, ε = 0.66, *p* = 0.0001, *η*_p_^2^ = 0.26). To further disentangle the activation patterns in the different time windows between the gestures, an additional statistical analysis was performed for each time window separately. Therefore, the gesture-only items were statistically investigated in 1-way ANOVAs, comparing the brain responses in all three conditions (blurred, pointing, give-me gestures) and, in a second step, the two recognizable gestures (without the blurred one) against each other. The first two time windows, 68–100 ms and 130–160 ms, did not show any significant differences between the three conditions (F(2, 48) = 1.88, *p* = 0.163). The earliest dissociation between gestures was observed in the third time window, 192–232 ms, (F(2, 48) = 22.99, ε = 0.75, *p < 0.0001*, *η*_p_^2^ = 0.48) with stronger responses to the give-me than to the pointing gesture, and pointing producing larger potential amplitudes than the blurred gesture (Fig. 2B). A further statistical analysis excluding the blurred gesture from the ANOVA confirmed the main effect of Gesture (F(1, 24) = 27.366, *p < 0.0001*, *η*_p_^2^ = 0.53) specific to the third time window.

To test for any repetition or exposure time effect (first vs second experimental blocks) during the experiment, an additional 2-way ANOVA was performed (Exposure time × Gesture) for each time window separately, hypothesizing that repetition might either contribute or diminish the differences between gesture types in the second experimental block. However, no significant interactions of the Exposure time factor were found. The second time window, 130–160 ms, did not show any significant differences between the gestures, while the third time window, 192–232 showed only a main effect of Gesture (F(2, 48) = 22.45, *p* < 0.0001, *η*_p_^2^ = 0.48).

#### Early effects, gesture-word combinations

The output of the 3-way ANOVA (Time window × Communicative function × Semantics) run on the gesture-word combinations showed a highly significant interaction between the factors Time window and Communicative function (F(2, 48) = 8.68, ε = 0.84, *p* = 0.0012, *η*_p_^2^ = 0.11). To further explore the differences between communicative functions in each time window, we ran separate 2-way ANOVAs (Communicative function × Semantics) for each time window, which revealed the following (see Fig. 2A): Whereas the first time window 68–100 ms did not provide for reliable differences between the two speech acts (F(2, 48) = 1.88, *p* = 0.163), the second window, 130–160 ms, revealed a significant main effect of Communicative function (F(1, 24) = 7.94, *p* = 0.0095, *η*_p_^2^ = 0.24). The third time window, 192–232 ms, once again gave evidence of a significant main effect of Communicative function (F(1, 24) = 12.82, *p* = 0.0015, *η*_p_^2^ = 0.34) with greater amplitude for request than for naming actions.

As in the analysis of the gesture-only condition, possible effects of gesture repetition or exposure time were addressed by an additional 3-way ANOVA (Exposure time × Communicative function × Semantics) run on each time window separately. These analyses failed to reveal significant main effects or interactions of the Exposure time factor, however indicating a non-significant tendency toward an interaction effect in the third time window (Exposure time × Communicative function, F(1, 24) = 4.01, *p* = 0.056).

#### Gesture-only vs. gesture-word combination

A further 3-way ANOVA (Time Window × Verbal context × Gesture type) was performed on the GFP amplitudes including both communicative function conditions and the recognizable gesture-only ones. This analysis revealed a significant interaction of Time window, Verbal context and Gesture type (F(2, 48) = 4.1, ε = 0.90, *p* = 0.025, *η*_p_^2^ = 0.14). When analyzing each time window separately, only the last one produced a significant interaction effect (F(1, 24) = 7.78, ε = 0.90, *p* = 0.010, *η*_p_^2^ = 0.24) showing a larger positivity only for give-me gesture compared to request function (p < 0.0001).

#### Late effects

The ANOVA performed on the predefined N400 window of the GFP failed to reveal any significant main effects or interactions. Notably, the differences between gesture-only conditions (F(2, 48) = 0.76, ε = 0.82, *p* = 0.45) or between the communicative functions of naming and requesting (F(2, 48) = 1.18, ε = 0.92, *p* = 0.31) were far from reliable. Likewise, the GFP data of trials with gesture-only items (again leaving aside the blurred gesture data) also failed to show any significant difference (F(1, 24) = 0.004, *p* = 0.94).

### Topographical ERP analysis

As only the second and third early time windows yielded significant differences in the GFP analyses, we included these in omnibus ANOVAs of ERPs from the gesture-only and communicative action conditions, respectively. Because the second peak was negative and lay at ~150 ms, whereas the third was positive and at ~210 ms, we call them ‘N150’ and ‘P210’ peaks, respectively. A 3-way ANOVA (Time Window × Gesture type × Topography) was performed for the gesture-only conditions and a 4-way ANOVA (Time Window × Communicative function × Semantics × Topography) for the Communicative function items. By these analyses, highly significant interactions were revealed for the gesture-only conditions involving the factors of Time Window, Gesture type and Topography (F(8, 192) = 25.5, ε = 0.46, *p* < 0.0001, *η*_p_^2^ = 0.51) and similarly for the Communicative function ERP responses between the factors of Time Window, Communicative function and Topography (F(4, 96) = 33.3, ε = 0.47, *p* < 0.0001, *η*_p_^2^ = 0.58). The additional results of the statistical analyses performed on each of the time windows separately are reported below. All significant planned comparisons reported below were still significant after application of Bonferroni correction. Figure 2C illustrates the main statistical findings for each time window and their topographical maps.

### N150 - time window 130–160 ms

There were no significant effects for the gesture-only conditions in this time window. However, the two-way interaction of Communicative function and Topography (F(4, 96) = 7.75, ε = 0.53, *p* = 0.00092, *η*_p_^2^ = 0.24) was revealed by the ERPs to gesture-word combinations, showing differences between topographical activation patterns between request and naming conditions. Pairwise Bonferroni comparisons confirmed significant differences at right posterior (*p* < 0.0001) electrodes.

To shed further light on language gesture interactions, an additional statistical analysis was carried out including both communicative acts and the corresponding gesture-only brain responses in a 3-way ANOVA, which revealed a significant interaction of Verbal context, Gesture type and Topography (F(4, 96) = 2.77, ε = 0.68, *p* = 0.047, *η*_p_^2^ = 0.10). This interaction was due to the topographical difference between linguistically embedded gestures yielding full speech acts and the absence of any significant neurophysiological dissociation between gestures presented outside linguistic context (see previous paragraph). The significant differences revealed by the Bonferroni planned comparison tests between naming and pointing conditions were found at left-posterior and central-midline (*p* < 0.0001) electrodes, and for request compared to give-me gestures at the left-posterior electrodes site (*p* < 0.0001).

### P210 - time window 192–232 ms

The gesture-only items showed a main effect of Gesture type (F(2, 48) = 6.5572, *p* = 0.00307, *η*_p_^2^ = 0.21) and a significant interaction of Gesture type and Topography (F(4, 96) = 31.91, ε = 0.58, *p* < 0.0001, *η*_p_^2^ = 0.57). This significant interaction was confirmed by the Bonferroni planned comparison tests that revealed significant differences between give-me, pointing and blurred gestures at the right- (*p* < 0.0001) and left posterior electrode clusters (*p* < 0.0001).

A main effect of Communicative function (F(1, 24) = 10.22, *p* = 0.00386, *η*_p_^2^ = 0.29) and a significant interaction of Communicative function and Topography (F(4, 96) = 31.07, ε = 0.65, *p* < 0.0001, *η*_p_^2^ = 0.56) was found for the gesture-word combinations. The Bonferroni planned comparison tests confirmed significant differences between the Communicative function conditions at several electrode sites: left anterior (*p* < 0.0001), central-midline (*p* < 0.0002), and right posterior (*p* < 0.0001).

The ANOVA carried out including both communicative acts, and the 2 recognizable gesture-only conditions revealed a significant interaction of Verbal context and Topography (F(1, 24) = 22.90, ε = 0.65, *p* < 0.0001, *η*_p_^2^ = 0.48) with larger positivity to gesture-only conditions compared to gesture-word combinations, especially at left and right posterior recording sites (*p < *0.0001). A further significant interaction of Gesture type and Topography (F(1, 24) = 32.54, ε = 0.64, *p* < 0.0001, *η*_p_^2^ = 0.57) revealed stronger positivity for the give-me gesture context carrying request information, as compared with the pointing gesture, especially at the central-midline (*p* = 0.004) and at left and right posterior (*p < *0.0001) electrode sites. No significant interactions were found between Verbal context and Gesture type (F(1, 24) = 0.001, *p* = 0.974) or between Verbal context, Gesture type and topography (F(4, 96) = 2.00, ε = 0.62, *p* = 0.1330).

### N400 – time window 300–500 ms

Somewhat contrasting with GFP results, statistical analysis of the N400-like component revealed significant effects. There was a significant interaction of Gesture type and Topography (F(8, 192) = 4.06, ε = 0.43, *p* = 0.006, *η*_p_^2^ = 0.14) for the gesture-only condition. The Bonferroni- planned comparison tests confirmed the significant difference at the left posterior electrode (p < 0.0001) sites only between give-me gesture and the blurred condition. Likewise, the communicative acts yielded a significant interaction between the factors Communicative function and Topography (F(4, 96) = 7.78, ε = 0.65, *p* < 0.0003, *η*_p_^2^ = 0.24), but this time the significant differences between naming and request functions were found at central-midline (*p* = 0.004) and in the left and right posterior (*p < *0.0001) electrode sites.

A third 3-way ANOVA was run incorporating both the communicative acts and the 2 recognizable gesture-only conditions revealed a main effect of verbal context (F(1, 24) = 78.33, *p* = 0.0014, *η*_p_^2^ = 0.76) confirming a larger N400 for gesture-word compared to gesture alone conditions and a significant interaction of Verbal context, Gesture type and Topography (F(1, 24) = 5.24, ε = 0.69, *p* = 0.0014, *η*_p_^2^ = 0.20). The Bonferroni planned comparisons confirmed significant topographical differences between request and give-me gesture and between naming and pointing gesture (Verbal context × Gesture Type × Topography interactions). Differences were prominent at the left and right anterior (*p* < 0.0001), central midline (*p* < 0.0001) and left and right posterior (*p* < 0.0001) electrode sites.

### Cluster-based permutation tests

To check for the robustness of our time-window specific analysis results above, cluster-based permutation tests were performed across a time range incorporating all time windows where significant differences between communicative actions (naming vs requesting) or gestures (give-me vs. pointing) had emerged from GFP and ERP analyses, that is, the N150 and P210 peak windows (overall window selected: 130–232 ms post-stimulus onset). The permutation test revealed one significant cluster in the distribution of give-me compared to pointing gestures in the time range of 210–230 ms (*p* < 0.025), consistent with GFP and ERP results reported above. In contrast, two significant clusters (in the distribution) distinguishing between the ERP distributions recorded in the request and naming condition(s): An early (one positive) cluster were found between 145 and 180 ms (*p* < 0.025), consistent with the N150 modulation to naming/requests revealed by the ERP analyses, and a subsequent cluster with opposite polarity from 210 to 230 ms (*p* = 0.005), which is again consistent with the positivity enhancement to requests relative to naming revealed by both GFP and ERP results (see topographical maps in Fig. 2C).

**Figure 3:**
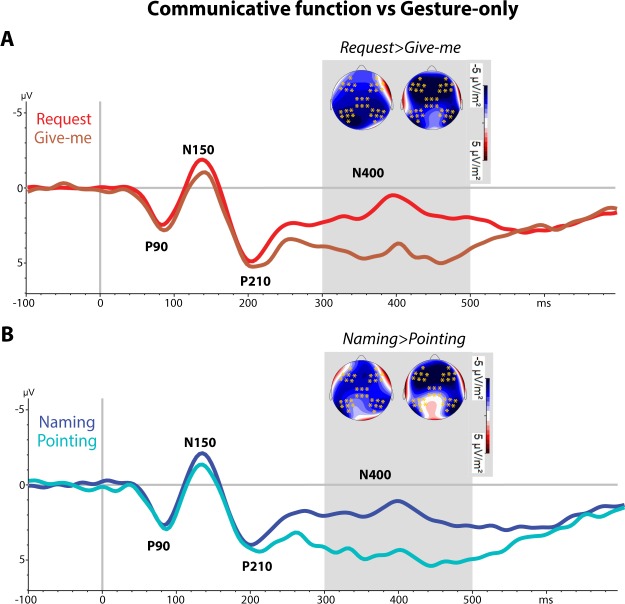
ERPs of Communicative function and Gesture-only conditions: (**A**) ERPs recorded in the request and give-me gesture-only conditions and (**B**) in those of naming and pointing averaged across 35 electrodes (see Fig. 1). The shaded regions highlight the time period (300–500 ms) where a significant cluster was found with the cluster-based permutation test and the brain at the top showing the different topographies (current source density, CSD) between the conditions and the orange asterisks marking the electrodes that survived the permutation test.

In addition, we performed an additional cluster-based permutation test over the time period of 300–500 ms to further investigate differences in the brain responses between the different conditions on the N400-like component. No significant cluster differentiating between the gesture-only condition (give-me vs pointing gestures) was found. Instead, one significant cluster (positive) on the distribution was found from 350 to 500 ms (*p* = 0.011), which distinguished between request and naming functions and was most pronounced at central-midline electrode positions. Furthermore, the permutation tests revealed highly significant clusters discriminating between naming function and pointing gestures and between request function and give-me gestures on the time period of 300–500 ms (*p* = 0.00019). The topographical distributions suggest a main contribution of the frontal-parietal-occipital electrode sites (see, Fig. 3).

### Source analysis

Source reconstructions were calculated for each subject and condition to estimate the cortical origin of the different neurophysiological responses. To evaluate differences in source strength or in the spatial distribution of sources across the brain between gesture conditions (pointing, give-me and blurred gestures) and between gesture-word combinations (naming and request), we performed a whole-brain voxel-wise paired *t*-tests only for those time windows that showed a significant difference in the statistical analyses of GFP, ERPs and cluster-based permutation tests described above.

For the speech acts including word and gesture combinations, the N150 time window, 130–160 ms, did not show any significant difference between conditions when applying whole-brain. However, within the P210 (time) window at 192–232 ms, sources of increased activation for requests compared to naming actions (*request* > *naming*) were seen in the frontocentral cortex which include the somatosensory and motor regions (BA 3/4, −28, −38, 58; 28, −38, 56, *p* < 0.001 uncorrected, *k* > 504, see Fig. 2A). For the gesture-only conditions, a significant cluster was obtained in occipital lobes, including bilateral extrastriate areas V2/3 (BA18/19, −40–82–4; 42 −74 −10, *p* < 0.001 uncorrected, *k* > 733, Fig. 2B), where give-me gestures elicited stronger sources than pointing (*give-me > pointing*). Similarly, a significant activation difference was found in the comparison between give-me and blurred gestures (*give-me > blurred*) within the occipital lobes, including bilateral extrastriate areas V2/3 (BA18/19, −30 −94 2; 24 −94 2, *p* < 0.001 uncorrected, *k* > 2000) and additionally in the frontocentral cortex including primary sensorimotor areas (BA 3/4, - + 24 −40 56, *p* < 0.001 uncorrected, *k* > 936). Furthermore, pointing compared to blurred gestures (*pointing > blurred*) showed a significant activation cluster only in the right extrastriate areas V2 (BA18, −34 −92 2, *p* < 0.001 uncorrected, *k* = 282; for details see Table 1). *T*-test against zero for gesture-word and gesture-only conditions (i.e., the two gestures in each condition were collapsed together) for the N150 and P210 latencies revealed activation of the left-perisylvian language and adjacent semantic areas including anterior temporal, parietal and motor regions (e.g., Binder *et al*.^56^, Pulvermüller 2013^57^, see Supplemental Material, Fig. S1). Notice the additional cortical areas activated for the gesture-word compared to gesture alone conditions.

**Table 1:** Whole-brain random effect analysis for the time window 192–232 ms.

	x	y	z	t-values	Nr. of voxels	*P*-values	Hemisphere	BA areas	Cortical areas
*Request > Naming*	−28	−38	58	3.61	529	0.001	L	3/4	somatosensory/ motor cortex
	28	−38	56	3.61	504	0.001	R	3/4	somatosensory/ motor cortex
*Request > Give-me*	−34	−86	−8	4.24	873	0.000	L	18/19	extrastriate cortex
	46	−66	−4	4.21	1440	0.000	R	18/19	extrastriate cortex
*Give-me > Pointing*	−40	−82	−4	4.25	733	0.000	L	18/19	extrastriate cortex
	42	−74	−10	4.12	1525	0.000	R	18/19	extrastriate cortex
*Give-me > Blurred*	−30	−94	2	6.60	2651	0.000	L	18/19	extrastriate cortex
	24	−94	2	5.04	4085	0.000	R	18/19	extrastriate cortex
	−24	−40	56	4.18	936	0.000	L	3	sensorimotor cortex
	24	−40	56	4.17	939	0.000	R	3	sensorimotor cortex
*Pointing > Blurred*	−34	−92	2	4.60	282	0.001	R	18	extrastriate cortex

For each significant contrast, the table shows MNI coordinates, t-values, number of voxels for each significant cluster, p-values (uncorrected), hemisphere, Brodmann labels and cortical areas.

**Figure 4:**
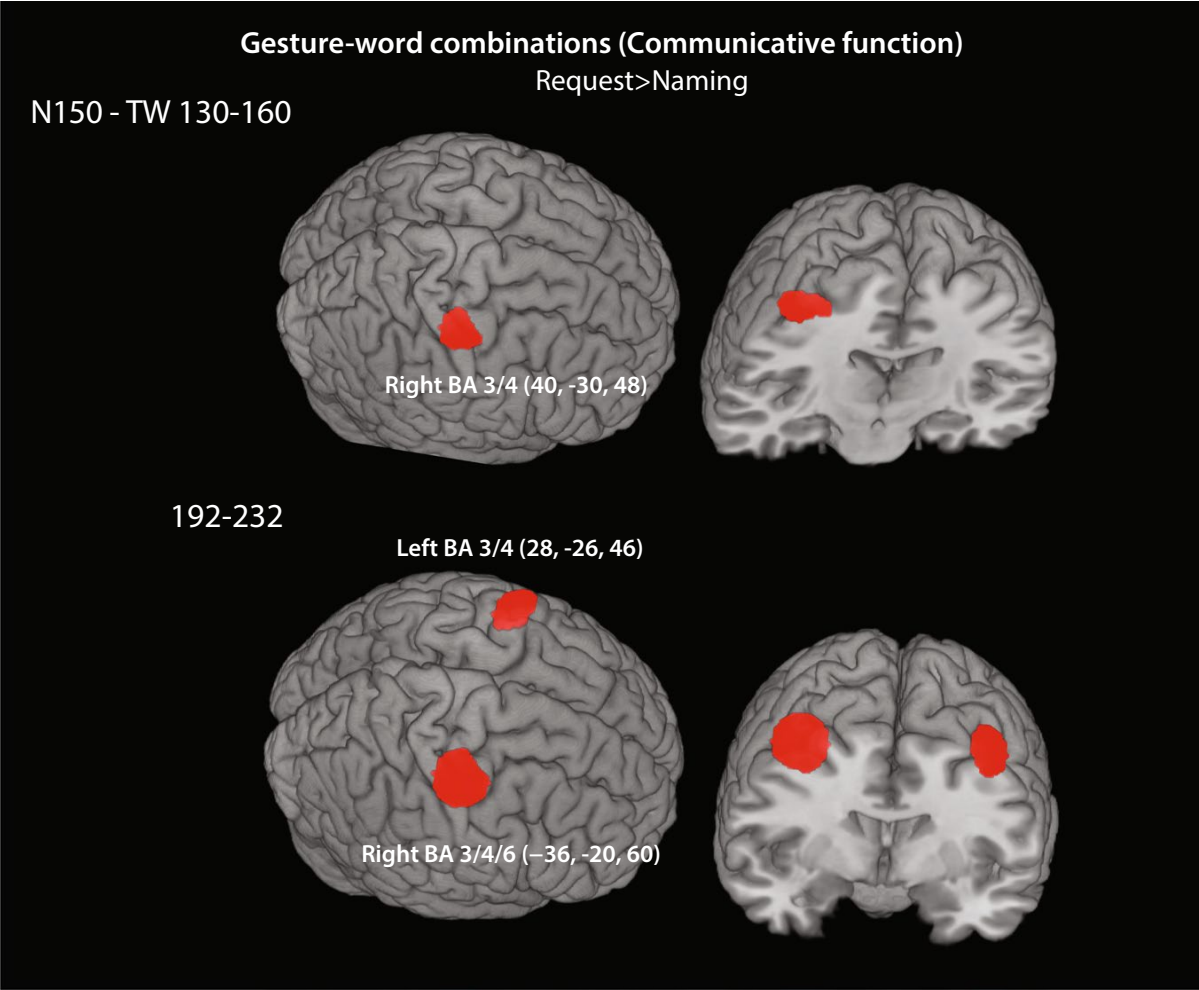
Source estimations in predefined regions of interest (ROI): Significant clusters of activation (p < 0.05, *FWE* corrected) where ROI-based analysis indicated stronger cortical sources for requests as compared with naming speech acts (*request* > *naming*) presented as gesture-word combinations; results are displayed for the time windows 130–160 ms (top panels) and 192–232 ms (bottom panels).

To better disentangle the patterns of activation revealed by the whole-brain analysis, we performed a second set of voxel-wise paired *t*-tests in predefined regions of interest using small volume corrections and guided by results from previous studies of speech act processing^34,35,40,55^ (for ROI definition, see Methods section). In this analysis, at latency 130–160 ms (N150) the contrast *request > naming* showed a significant cluster activation in the right sensorimotor areas (BA 3/4, 40, −30, 48; *p* = 0.038, FWE corrected, *k* = 158, Fig. 4 – top panels). The same pattern of activation was also revealed in time window 192–232 ms (P210), but now in left sensorimotor areas (BA3/4, −28, −38, 58; *p* = 0.037, FWE corrected, *k* = 243, Fig. 4 – bottom panels) and in right sensory, motor and Supplementary Motor (SMA) cortices (BA3/4/6, −36, −20, 60; *p* = 0.024, FWE corrected, *k* = 832, see Table 2). The other paired *t*-tests did not show any significant differences.

**Table 2:** ROI analysis for the contrast request > naming for the time windows 130–160 and 192–232 ms.

	x	y	z	t-Values	Nr. Of Voxels	*P*- FWE corrected	Hemisphere	Brodmann areas	Cortical areas
*N150 Request > Naming*	30	−28	44	3.4	178	0.035	R	3/4	somatosensory/ motor cortex
*P210* *Request > Naming*	28	−26	46	3.61	832	0.024	R	3/4/6	somatosensory/ motor cortex/SMA
	−36	−20	60	3.51	243	0.037	L	3/4	somatosensory/ motor cortex

For each significant contrast, the table shows MNI coordinates, t-values, number of voxels for each significant cluster, FWE-corrected p-values, hemisphere, Brodmann labels and cortical areas. The other possible contrasts did not produce any activations that were significant at FWE-corrected p* < *0.05 threshold.

## Discussion

To determine the time range when human subjects understand and discriminate between different communicative functions, we investigated the brain signatures of speech act understanding expressed by a combination of simultaneously presented gestures and written words. We found early and distinct neurophysiological correlates of communicative function understanding. Requests, expressed by an open-hand gesture (the give-me gesture) appearing together with a meaningful word, and naming actions, performed by using a pointing gesture together with a word, elicited different event-related potential amplitudes and topographies as early as 130–160 ms (N150 response), and also slightly later around 210 ms (192–232 ms; P210 response). In contrast, the gestures presented on their own, without any referential linguistic context, failed to show comparably early neurophysiological dissociations, and became neurophysiologically distinct only in the later time window, at 192–232 ms. Neurophysiological source localisation of the distinct activation patterns revealed increased activity in sensorimotor regions for requesting compared to naming functions, whereas the sources for the comparison between give-me and pointing gestures were localized in posterior visual areas of the cortex. P210 and N400-responses tended to be more negative-going for full communicative acts composed of gestures and words compared with the same gestures presented alone (outside verbal context), which may suggest prolonged semantic/pragmatic integration of information provided by gestures and words. This latter observation is in line with previous neurophysiological studies on iconic and symbolic gesture understanding showing an N400 deflection reflecting gesture-language integration^2,7,9–11,58^.

These effects are the first to show that two ontogenetically most basic communicative functions — naming and requesting expressed by an interplay between gestural indexes and linguistic symbols — are processed instantaneously in the human brain. The neurophysiological dissociation between the gestural-verbal communicative acts of naming and request at 130–160 ms provides an upper limit for the onset of neurocognitive differences discriminating between the communicative functions of these actions. Importantly, the fast processing of communicative functions cannot be due to the verbal materials employed, as these were exactly balanced between naming and request conditions, and cannot be due to any physical or cognitive differences between the gestures either, as gestures alone produced a neurophysiological dissociations only later (from around 200 ms). The early neuropragmatic processes were only present when information about both the propositional content and the illocutionary function of the communicative action was provided so that the speaker’s communicative intentions were fully transparent and interpretable to the partner. Their ready and quick differentiation at the neurophysiological level is consistent with an early and fundamental role of pragmatic information in language processing and specifically with the prominent pragmatic role of hand gestures^19^ forming an integrated communication system with spoken language^1,7,59^.

The fast processing and integration of pragmatic and semantic information in understanding communicative acts composed of words and indexical gestures agree with many previous studies on human complex language and action understanding^46,60–65^, and face recognitions^66–68^. For instance, Proverbio and colleagues^62^ reported an N170 when subjects were watching hundreds of different goal-directed actions between 2 persons, revealing fast coding of human action understanding. Here, we show comparably early brain dissociations between indexical gestures presented in verbal context and carrying distinct communicative functions.

**Figure 5:**
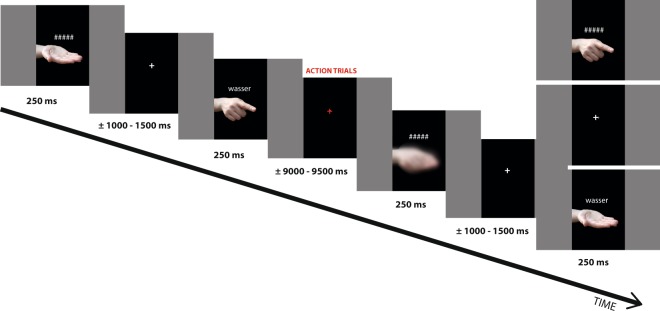
Schematic illustration of task and stimulus presentation sequences. The depicted gestures (gesture-word combination, gesture-only and blurred condition) were presented randomly for 250 ms with an inter-stimulus interval (ISI) of 1000–1.500 ms. Participants were asked to attend to and try to understand the displayed speech acts and gestures. When the fixation cross turned red, thereby signalising a catch trial and the need to respond, participants had to identify the card depicting the referred object (18 cards of different objects were spread over the table) and put it into a box after a request, or point to the object depicted on the card after a naming speech act.

Alternative traditional accounts that envisaged later-stage processing of pragmatic information following phonological, lexical, syntactic and semantic knowledge access^41–43,69^. In particular, our results showed an early interaction of the pragmatic aspect of communicative function with the semantic category of the verbal materials, thus not only demonstrating simultaneity of pragmatic and semantic information processing but also suggesting that such processing may be interactive. These results conflict with models proposing serial semantic and pragmatic processing stages or stepwise ‘semantic’ and ‘message’ levels of linguistic information access (Friederici, 2002^41^, 2011^42^, Fig. 1 and Fig. 11 respectively, see also Pickering & Garrod, 2004^43^, 2013^69^, see Figs 2 and 5 respectively). Instead, our present data are consistent with current positions that emphasize the near-simultaneity of pragmatic processing with other aspects of the understanding process, along with a fundamental role of pragmatic information in language acquisition, evolution, and use^46,48–51,70^.

Intriguingly, the source localisation analyses revealed different neural activations for distinct communicative functions. We propose that these in part, reflect the specific pragmatic knowledge retrieved in the understanding of these communicative acts. In particular, some features of the expected partner actions commonly following a given speech act may become manifest in the brain indicators of that speech act. Consistent with this proposal, the expectation that, upon understanding a typical request, the partner will grasp an object and hand it over to the speaker may underlie the relatively enhanced activity of sensorimotor regions in request understanding. This perspective implies a ‘social-pragmatic response’ in request processing, according to which brain regions important for the processing of predicted partner actions and the associated commitments^71–73^ are part of the cortical network activated in request processing. Below we discuss our findings in more detail in light of previous studies and linguistic-pragmatic theories, and in view of future research perspectives.

The stronger engagement of frontoparietal areas, including sensorimotor regions, for the communicative request function related to the intrinsic pragmatic action knowledge of requests is consistent with recent studies of speech act comprehension employing more precise localisation tools (fMRI). In particular, Egorova *et al*., (2016) found activation in hand-motor cortex and in Broca’s area to be significantly stronger when understanding requests compared to naming actions presented as single word utterances^40^. Other studies investigating indirect replies and indirect request functions documented activation in areas implicated in mentalizing, action planning and motor control^34,35^. Similarly, Bašnáková *et al*., (2014) carried out a fMRI study contrasting direct and indirect replies to questions^74^. Activation of indirectness contrasted to directness was found in Theory of Mind areas (the ability to attribute mental states to oneself and others) and in motor regions for action processing. All these findings including the present one provide strong indication that the general communicative function of requesting involves frontal parietal and likely, sensorimotor cortices, which is consistent with the role of the motor system in action processing during comprehension and production of verbal description of actions^75–80^.

The present early neurophysiological indexes of communicative function processing are consistent with those of a previous EEG study of speech act understanding performed with single words used to name or request objects in matched videotaped scenes^54^. In that study, the earliest neurophysiological differences between the two speech act types were found ca. ~150 ms after critical (written) word onset, thus exactly matching our present results. A further similar MEG study^55^ indicated even earlier speech act discrimination around ~50 ms, a finding which we could not confirm here. However, as mentioned in the introduction, these and similar earlier studies used a blocked presentation of speech act types, displaying a context sentence before the critical utterance (‘What are these called?’ for naming and ‘What would you like?’ for requests), which determined the upcoming speech act seconds before the critical linguistic stimulus appeared. Therefore, the reported earliness of speech act specificity in the brain responses may, in part, have been due to the predictability of illocutionary force from previous context^54,55^. Our present study was not subject to this potential bias and caveat. To exclude the possibility that any pre-existing information about upcoming speech acts could potentially speed up the recognition and understanding process, while at the same time keeping constant the utterances carrying pragmatic-communicative function, a different research strategy was necessary. Here, we presented the same referent words in the context of, and simultaneously with, gestures, thus providing all information about the speech act, propositional and illocutionary, exactly at stimulus onset. This excludes a bias of pre-stimulus context information toward speeding up neuropragmatic responses.

Although both speech act types were presented in randomized order, a possible predictability effect based on gesture and speech act repetition could have developed over time across the experiment. It is possible that this relatively good predictability of speech acts and gestures may have speeded up the neural responses of communicative action understanding beyond normal processing. However, such speeding due to repetition would predict that, only with progression of the experiment and resultant routinization, the early brain responses indexing speech act comprehension might have developed. However, additional statistical analysis did not reveal any significant interactions of speech act or gesture type with exposure time (first vs second experimental block) in the N150 time window. Hence, these results, together with the main effect of communicative function, confirm the general presence of early brain indexes of communicative function processing expressed by the combination of gesture and language. A non-significant tendency toward a time effect was only seen for the later window of the P210.

We also note that, in the current study, a disproportion of female participants took part in the experiment. Previous empirical studies reported sex difference in the neural amplitude responses during the processing of social information^81^ and also for early gestural acquisition in female compared to male infants^82^. Hence further work should investigate possible gender differences in the neural signatures of communicative actions understanding.

Interestingly, when subjects were presented with the gestures alone, which denote communicative function or speech act type, but no semantic-referential information, they showed a neurophysiological dissociation at a later stage than when the speaker’s intentions were fully expressed by the combination of gestures and words. Specifically, at latency 192–232 ms, where a posterior P2-like positivity developed in the event-related potential, the give-me gesture indexing the illocutionary force of a request produced stronger activation than the pointing gesture indicative of naming. In contrast, the blurred gesture, which resembled both hand gestures to the same degree and did not convey any information about illocutionary force or referential content, failed to elicit a clear peak in its ERP (see GFP waveforms Fig. 1). On first view, one may argue that the subjects’ task, which focused on trials with gesture word combinations, could have biased subjects to process these more quickly than the gestures presented on their own. On the other hand, no instructions were given to that end, and, for subjects to realize that catch trials appeared subsequent to gesture word combination trials, some substantial time of the experiment would have elapsed. In this context, it is important to recall the lack of neurophysiological dynamics throughout the experiment, with ‘repetition’ analyses failing to yield significance, both for the gesture-only and for the combination trials. If subjects had realized over time that gesture only trials were less relevant and therefore had processed them differently so that ERP signatures got delayed, we would have expected a change in the neurophysiological signatures between early and late gesture-only trials. As no evidence for such temporal change was discovered, we suggest that the 150 ms vs 210 ms latencies of the earliest gesture-word and gesture-only manifestations in physiology reflect a true difference in cortical processing. Hence, these results show that the earliest neurophysiological differentiation, around ~150 ms, between communicative functions carried by gestural-verbal interplay cannot be explained by physical features of the gestures alone, or by the cognitive processes these features give rise to. Further statistical testing of the event-related potentials from the second time window around ~150 ms revealed a statistically significant interaction of Verbal context [gesture-word combination vs gesture-only], Gesture type [pointing vs give-me], and Topography [five levels: LA, RA, CM, LP, RP], which was due to the presence of a difference for the language-embedded conditions contrasting with an absence thereof for the gesture-only trials (see result section for more details). This provides evidence that the early speech act comprehension effect shown by our data is related to language-gesture interplay.

The gesture-only conditions served as control conditions for the linguistic, gesture-embedded language conditions. However, it is important to note that, for these gesture-only conditions, a contribution of physical features on the differential brain responses is difficult to exclude. However, a second, alternative possibility for interpretation exists, namely that it is indeed the distinct pragmatic information immanent to the gestures that are reflected neurophysiologically. In this view, the attention-directing function and illocutionary force typically associated with these hand gestures may be relevant, as reported by previous studies (e.g. see Gunter *et al*.^83^). Whereas pointing is typically used to attract attention to an object, the give-me gesture is used to draw attention to an object and to ask for it^20–23^. Although gestures alone can carry pragmatic information about the illocutionary force, the communicative actions of request and naming also require propositional referential content to become functional; it must be clear which item is requested or named. In turn, the absence of the referential content in the gesture alone conditions showed a smaller N400 response compared to the gesture-words conditions. Notice that previous studies (e.g., Gunter and Bach 2004^4^) documented an N400 effect for gesture presented alone, however, as mentioned in the introduction, these studies have investigated emblematic gestures that have a specific semantic meaning typically used to replace speech (e.g., thumb up). Hence an increase of the N400 response for this type of gestures is indeed expected^4^. Here, we show that this is not the case for deictic communicative gestures presented outside language context. Notice that a recent study^83^ reported an N400 effect for inconsistent use of abstract pointing understanding. This result may be due to the unexpected appearance of the gesture in the ‘inconsistent’ use and therefore fit into the typical N400 interpretation in terms of semantic expectancy and cloze probability (e.g., Kutas and Federmeier, 2011^84^, 2000^85^). This said, we also note that our own data are open to this interpretation. In the present experiment, highly predictable gestures appeared on their own, and no N400 may, therefore, have appeared due to good gesture-predictability. However, word gesture conditions included a predictable gesture and an unpredictable meaningful word, thus giving rise to a semantic surprise effect.

A further and fundamental difference between this and earlier work on the understanding of communicative actions needs to be mentioned: Previous works^38–40,54,55,61^ investigated the neural mechanisms of speech act comprehension by studying participants who observed dialogues in a 3^rd^ person perspective and thus *did not partake* in the interaction. A further crucial novelty of the present study lies in the fact that experimental subjects took on the *role of the partner participating in social interaction* to whom speech acts were addressed (2^nd^ person perspective). All gestures and gesture-word combinations were directed toward the participants and, in some of the trials, participants had to respond correctly to the speech acts (see methods for more details). The observed parallelism in speech-act-discriminating cortical sources across 3^rd^ and 2^nd^ person perspective experiments increases the trust in these results. In particular, the relatively engagement of sensorimotor areas in request comprehension possibly signaling the richer action sequence structure of requests (as compared to naming) appears to be independent of perspective^40^. However, future studies should investigate communicative functions in 1^st^, 2^nd^ and 3^rd^ person perspective in a more systematic manner and possibly in more natural communicative settings, in which, for example, real objects are named or requested by a confederate with real social-communicative interaction between partners. Still, such natural settings come at a cost, as experimental parameters are more difficult to control. Furthermore, it is important to emphasize that here we explored the comprehension of communicative acts with that of gestures presented in isolation. However, other body movements, including body language involving all extremities, facial expressions, preparatory lip movements and eye gaze are all relevant as non-verbal cues in communication (e.g. Proverbio *et al*.^81^). Looking at iconic and indexical gestures can therefore only represent a first step in the exploration of bodily communicative information, which calls for further investigation.

## Conclusion

The present electroencephalography (EEG) experiment investigated the neural signature of communicative functions emerging early in language development, the naming and requesting function carried by gestures and words. Based on the present EEG results, three main conclusions can be drawn. First, communicative function is processed very quickly in the human brain. Brain responses indexing different communicative functions (requesting and naming) differed clearly within ~150 ms after stimulus presentation, thus providing an early upper limit for the onset of pragmatic understanding processes. Such fast processing of pragmatic information in communicative acts, which occurred simultaneously with the earliest semantic effects, provides strong support for parallel processing models of linguistic information in the mind and brain and argues against serial and cascade processing models of language understanding postulating 100 s of milliseconds between phonological access and lexical, syntactic, semantic and finally pragmatic processes. The observed earliness and simultaneity sit comfortably with the general importance of pragmatic or communicative function information in language processing. Second, processing of the gestures alone (give-me, pointing and blurred gestures) without any referential-semantic information showed relatively late activation differences, at ~210 ms, thus proving that the early speech act difference arising ~60 ms earlier cannot be explained by differences between the gestures, but must be due to linguistic-gestural interplay of information. Third, EEG source analysis provides evidence for distinct neural activation for different communicative acts expressed by gesture-word combinations. Examining these, the relatively stronger brain activation seen in the evoked potentials of acts of requesting compared to naming can, at least in part, be traced back to activation of frontocentral including sensorimotor regions, which may carry the processing of richer action-communication knowledge relevant for understanding and producing requests.

## Methods

### Participants

Twenty-six healthy right-handed volunteers (mean age 26; range 19–35; 21 females) took part in the study after giving informed written consent. All participants were monolingual native speakers of German with normal or corrected to normal visual acuity and had no record of neurological or psychiatric disease. Participants were paid for taking part in the experiment, and their right-handedness was confirmed by the Edinburgh Handedness Inventory^86^ (mean laterality quotient ±82, 4.2 SE). Procedures were approved by the Ethics Committee of the Charité Universitätsmedizin, Campus Benjamin Franklin, Berlin, Germany and this research was performed was carried out in accordance with the aforementioned regulations. All participants signed an informed consent form prior to the start of the experiment.

### Stimuli and procedure

Photographs of pointing and give-me hand-gestures were used as experimental stimuli indexing the illocutionary roles of naming and requesting communicative acts^87^. The depicted gestures were presented simultaneously with single written words, which provided the referential/propositional information. Gesture-word combinations were shown in the centre of a screen (see Fig. 5). 156 German nouns were selected from three semantic categories referring to either *tools, food items* or *animals* (52 words per category). Similar to previous studies^88,89^, these semantic categories were confirmed by semantic ratings, where subjects judged the semantic relatedness of each word to *hand* and *mouth* actions and *visual-perceptual* features using Likert scales ranging from 1 (no relation) to 7 (very strong relation). Ratings were obtained from 20 German speakers (mean age 20 years; range 18–28 years) not involved in the neurophysiological experiment. All semantic categories were subsequently matched for a range of lexical and sub-lexical psycholinguistic variables, as revealed by the DLEX corpus^90^. The lexical stimuli were matched for a range of psycholinguistic features, including word length, number of syllables, phonological stress, normalized lemma frequency, character bigram frequency, character trigram frequency, and initial character-, initial character bigram-, and initial character trigram-frequency, as well as for number and corpus frequencies of orthographic neighbours. F-Tests failed to indicate any significant differences between the three categories on these variables (for details see Table 3).

**Table 3:** Matching of psycholinguistic properties between semantic categories.

	Animals		Foods		Tools			
	*M*	*SE*	*M*	*SE*	*M*	*SE*	*F*	*p*
Lemmafrequency p. Mio.	6,25	0,77	6,38	1,46	6,11	0,91	0,02	0,984
Number of Syllables	1,75	0,06	1,81	0,06	1,90	0,04	2,15	0,120
Length	5,81	0,21	5,88	0,23	5,94	0,20	0,10	0,905
Cumulated Character-Bigram Frequency p. Mio.	260391	16443	209463	17833	254403	19861	2,37	0,097
Cumulated Character-Tigram Frequency p. Mio.	157934	10029	117739	11548	139712	13272	2,96	0,055
Initial CharacterFrequency p. Mio.	14617	805	15499	844	15310	1044	0,26	0,768
Initial Character-Bigram Frequency p. Mio.	2471	255	2045	278	2411	297	0,69	0,502
Initial Character-Tigram Frequency p. Mio.	819	250	535	186	858	251	0,58	0,561
Cumulated Frequency of Coltheart Orthographic Neighbours p.Mio.	52,75	31,92	82,74	60,19	53,74	16,15	0,18	0,837
Number of Orthographic Neighbours (Coltheart N)	5,71	0,80	6,28	1,09	7,13	0,80	0,62	0,541
Cumulated Frequency of Levenshtein Orthographic Neighbours p.Mio.	64,00	32,47	188,43	101,61	60,05	16,36	1,37	0,257
Number of Orthographic Neighbours (Levenshtein N)	8,45	0,99	9,07	1,38	10,13	0,95	0,57	0,565
Relation to visual sensation	6,25	0,77	6,38	1,46	6,11	0,91	0,85	0,431
Arm-Action relatedness	1,27	0,05	2,16	0,07	5,57	0,09	1000,33	p < 0.001
Face-Action relatedness	1,44	0,10	5,14	0,06	1,26	0,09	669,35	p < 0.001

*p* values denote results from a one-way analysis of variance with the factor semantic for the respective variables.

Each of the overall 156 words was presented once in each of two ‘speech act conditions’, where it was co-presented with a gesture, either with the pointing gesture to yield a naming action or with the give-me gesture to produce a request. To separate the contributions of gestures and words to the brain response, the pointing and give-me gestures (52 each) were presented outside linguistic context in ‘gesture-only conditions’. Instead of words, a meaningless strings composed of hash marks and matched in length to the word sample (e.g. ‘####’) was co-presented with the gesture, so as to provide a control condition for studying gesture processing outside linguistic context, with the hash mark strings controlling for the additional visual stimulation. As a further ‘gesture control’ condition, an unrecognisable blurred gesture picture was co-presented with hash mark strings, so that subjects were not able to identify either the illocutionary roles of the speech acts expressed through the gesture or the referential information provided by the utterance; the blurred gesture-hash mark combinations were also presented 52 times.

The experiment consisted of two experimental blocks, whose order was counterbalanced across subjects. Each block included 246 trials, 156 of them comprising all vocabulary items, each assigned to one of the speech act conditions; the two blocks used complementary assignments of words to speech acts and different stimulus randomizations. The presentation was controlled by E-prime 2.0 (Psychology Software Tools, Pittsburgh, PA). Each trial started with a fixation cross presented in the middle of the screen, followed by stimulus presentation for 250 ms. The inter-stimulus interval (ISI), during which the fixation cross was visible again, varied randomly between 1000 and 1.500 ms (Fig. 5). The experiment was conducted in the electrically and acoustically shielded chamber of the Brain Language Laboratory at the Freie Universität Berlin. Inside the chamber, a computer screen was used to present the stimuli to the participants, who were seated 80 cm away from the monitor. Stimuli were constructed and presented in such a way that the participants were the ‘communication partners’ of an unknown speaker and had to understand – and sometimes react to – his/her communicative acts. To involve participants in the dialogues and to keep their attention, ca. 13% (12) of the catch trials were presented, randomly interspersed within each block. A red fixation cross after a stimulus signaled the catch trials (which lasted for 9000–9.500 ms), so that only after stimulus perception the subjects new whether to react or not. Specifically, for the catch trials, 18 cards depicting different objects were spread over the table. After being shown the red fixation cross, participants had to respond to the previous communicative act appropriately: to requests by grasping the appropriate card and putting it into a box positioned in front of them, and to the naming speech acts by pointing to the card depicting the named object. A web camera was positioned in the upper part of the computer screen, and the subjects were informed that a confederate was observing their reactions on the communicative functions. The confederate was also meant to be the recipient of the box content in the request condition. Response accuracies were recorded; brain responses in catch trials were not analysed.

### EEG recording

The EEG was recorded through 64 active electrodes embedded in a fabric cap (the green and yellow subsets of electrodes from the actiCAP 128Ch Standard-2; Brain Products GmbH, Munich, Germany) with the following modifications: the reference was moved from FCz position to the tip of the nose, the electrode occupying the PO10 position replaced the empty FCz position. The PO9 and FT9 electrode positions were reassigned as EOG channels placed below and above the left eye respectively and the FT10 electrode to the right outer canthus to measure the vertical and horizontal electro-oculograms. All electrodes were referenced to an electrode placed on the tip of the nose. Data were amplified and recorded using the Brain Vision Recorder (version: 1.20.0003; Brain Products GmbH), with a pass band of 0.1–250 Hz, sampled at 500 Hz and stored on disk. Impedances of all active electrodes were kept below 10 KΩ.

### EEG data processing

The following stages of pre-processing were carried out in EEGLAB 13.4.3b^91^. Data were down-sampled offline to 250 Hz and low pass filtered at 30 Hz. To obtain the vertical EOG, the difference between upper and lower left eye electrodes was calculated, and the horizontal EOG was computed from the average of the latter two minus the potential at the right outer canthus. EEG channels containing no signal or substantial artefacts were rejected after visual inspection. Independent component analysis (ICA) was used to derive 35 components from the data. Components that correlated with either vEOG or hEOG with *r* < −0.3 or *r* > 0.3 were removed from the data, thus significantly reducing eye-related artefacts^92–94^. Removed channels were then spherically interpolated back into the data. Subsequently, the EEG data were segmented into epochs starting 100 ms before the onset of the stimuli and ending 700 ms after it. For baseline correction, the 100 ms pre-stimulus interval was used. Epochs with signals exceeding −100 and 100 µV were discarded and only subjects with a rejection rate below 20% were included in the final statistical analysis. Using this criterion, one data set was rejected because of the poor quality of the recordings (due to line noise on signals and too low impedances during the recordings). Therefore, data from twenty-five subjects entered the EEG analysis. In this sample, the average trial rejection rate was 3.6%.

### Data analysis

#### GFP analyses

To determine the amplitude and peak latencies of the evoked potential components, the Global Field Power (GFP) waveform was computed across all electrodes, subjects and conditions (Fig. 1). GFP is plotted as a function of time and displays the rectified overall activity across the scalp. The time windows of interest were defined around GFP peaks; window widths were determined using the Full Width at Half Maximum (FWHM). To quantify any global effects of, and differences between, communicative function (gesture-word combinations) and gesture-only conditions (pointing, give-me and blurred gestures), an initial statistical evaluation was performed on the GFP mean amplitudes across these time windows. To this end, a 2-way repeated-measures Analysis of Variance (ANOVA) was run on gesture-only items with the factors Time window [three level: TW1 vs. TW2 vs. TW3] and Gesture type [three levels: pointing, give-me vs. blurred gestures], and, for planned comparison testing, additional 1-way ANOVAs were performed for each time window with Gesture type as the only factor.

Similarly, for the gesture-word responses a 3-way ANOVA was performed including the factors Time window [three levels: TW1 vs. TW2 vs. TW3], Communicative function [two levels: naming vs requesting] and Semantics [three levels: tools vs. foods vs. animals]. We further performed a second statistical analysis on each time window separately with the factors Communicative function and Semantics as described above. In addition, a 3-way repeated-measures Analysis of Variance (ANOVA) was run including both gesture-word conditions and 2 of the 3 gesture-only responses (now omitting the blurred gesture) with the factors Time Window [three level: TW1 vs. TW2 vs. TW3], Verbal context [two levels: gesture-word combination vs. gesture-only] and Gesture type [two levels: pointing vs. give-me gesture]. Subsequently, each time window was further analyzed separately.

To test for any effects of frequent repetitions and exposure time across the experiment, an additional 3-way ANOVA was performed on the gesture-word data of each time windows with factors Exposure time [two level: first vs. second experimental block], Communicative function [two levels: naming vs. requesting] and Semantics [three levels: tools vs. foods vs. animals]. The same statistical analyses were also performed on the gesture-only items but omitting Semantics as a factor.

An additional N400 time window was selected 300–500 ms (e.g., Kutas and Federmeier, 2011^84^) and treated separately. To this end, three different ANOVAs were performed to test for any significant differences in the N400 or any N400-like component. We applied a one-way ANOVA on the gesture-only GFP data with Gesture type as the only factor [three levels: pointing vs. give-me vs. blurred gestures], a 2-way ANOVA on the gesture-word combinations GFP data with the factors of Communicative function [two levels: naming vs. requesting] and Semantics [three levels: tools vs. foods vs. animal] and additionally, a 2-way ANOVA including both communicative function and gesture-only conditions with the factors Verbal context [two levels: gesture-word combination vs. gesture-only, i.e., pointing vs. give-me gestures] and Gesture type [two levels: pointing vs. give-me gesture].

#### ERP analyses

The GFP evaluations described above were complemented with an analysis of event-related potentials from selected electrodes focusing only on the latencies that showed a significant difference in the GFP evaluation described above. To this end, we chose a representative sample of 35 electrodes placed at left anterior (LA: AF7, F7, F5, F3, FT3, FC5, FC3), right anterior (RA: AF8, F4, F6, F8, FC4, FC6, FT8), central-midline (CM: FCz, C1, Cz, C2, CP1, CPz, CP2), left-posterior (LP: TP7, CP5, CP3, P7, P5, P3, PO7) and right-posterior (RP: CP4, CP6, TP8, P4, P6, P8, PO8) (see inset in Fig. 1). A 4-way ANOVA was performed with the factors of Time window [two levels: TW2 vs. TW3], Communicative function [two levels: naming and requesting], Semantics [three levels: tools, foods and animals], and Topography [five levels: LA, RA, CM, LP, RP]. Additional statistical analyses were performed on each of the time windows separately (including also the N400-like responses, which was treated separately) with the factors of Communicative function and Semantics. The same statistical analyses were also performed on the gesture-only items but omitting Semantics as a factor. Additionally, to explore any significant difference in amplitude/activation between both communicative function and gesture-only conditions, a 3-way ANOVA was carried out with the factors Verbal context [gesture-word combination vs. gesture-only], Gesture type [pointing vs. give-me], and Topography [five levels: LA, RA, CM, LP, RP] for each of the time windows. Greenhouse-Geisser correction^95^ was applied when sphericity violations were found. Corrected p-values along with epsilon (ε) values are reported throughout. Partial eta-square (*η*_p_^2^) values are also stated, which is defined as an index of effect size (0.01–0.06 small, 0.06–0.14 medium and >0.14 large^96^).

#### Cluster-based permutation tests

To further quantify between condition differences in brain responses by avoiding the problem of multiple comparisons^97^, we applied a (non-parametric) cluster-based Monte Carlo permutation test to the ERP data as implemented in the FieldTrip toolbox for MATLAB^98^. These cluster-permutation tests were computed over the 35 frontal, parietal and occipital electrodes (the same applied for the previous described topographical analysis, Fig. 1) by randomly exchanging data between the different conditions and producing the maximal positive and negative cluster of each permutation (5000 permutations). Based on this, the clusters were deemed significant if the likelihood of occurrence was below *p < *0.05 two-tailed (0.025 for each tail).

#### Source localization

To identify the cortical origin of the neurophysiological responses underlying the differences between conditions (communicative function and gesture-only conditions), we performed distributed source localization analyses. We used the structural MRI included in SPM12 to create a cortical mesh of 8196 vertices, which was then co-registered with each subject’s electrode cap space using 3 electrodes as fiducials: FP1, TP9, and TP10. The volume conductors were constructed with an EEG (3-shell) boundary element model. The method used for source estimation was the multiple sparse prior (MSP) technique, specifically the ‘greedy search’ algorithm^99^, which had previously been used in our lab (e.g., Grisoni *et al*.^100^). Each response, within its respective time window, was then inverted for each subject, thereby constraining spatial source solutions uniformly across participants^101^. Activation maps were then smoothed using a Gaussian kernel of FWHM 12 mm, resulting in 5 images per participant (i.e., 2 for the communicative conditions naming and requesting and 3 for the gesture-only items, pointing, give-me, and blurred gestures). Source averages and statistics were calculated at the group level, and only on those latencies where significant effects between conditions were found in the statistical analysis of event-related potentials.

To evaluate potential differences in source distribution between the conditions across the whole brain, we carried out voxel-wise paired *t*-tests. For the whole-brain source analysis, clusters that survived to the threshold of *p* < 0.001 (uncorrected) were considered significant if they were larger than *k* > 10 voxels (i.e., cluster-extent based thresholding^102,103^). We further performed a second set of paired *t*-tests for predefined regions of interest (ROIs) based on previous studies of speech act processing^34,35,40,55^. The ROIs included (i) the left inferior frontal gyrus (IFG) relevant for action-semantics and the mirror neuron system system^104,105^, which has been found active in request comprehension^40,55^, and (ii) the dorsolateral central motor areas, where hand movements are controlled (taken from a finger movement localizer^89^ and previous work had shown similarly prominent activation during Request understanding^34,35,40^). ROIs were created with Marsbar 0.44 (MARSeille Boîte À Région d’Intérêt, SPM toolbox) as 12-mm-radius spheres (i.e., matching the FWHM of the smoothing parameter) centred at the above-mentioned regions. These ROIs were then combined in a unique mask used as Explicit Mask in the voxel-wise paired t-test design. For this analysis, *P* values were thresholded more conservatively (*p* < 0.05 after family-wise error (FWE) correction).

## Supplementary information

Supplemental Material

## Data Availability

The datasets generated during the current study are available from the corresponding author on reasonable request [R.T.].
