# Supplementary material for: Neurophysiological evidence for rapid processing of verbal and gestural information in understanding communicative actions
Source: Sci Rep. 2019 Nov 8;9:16285. doi: 10.1038/s41598-019-52158-w (PMC6841672; doi:10.1038/s41598-019-52158-w)
Supplement: Supplementary file 1 — Supplemental Material [file 41598_2019_52158_MOESM1_ESM.pdf]

Supplemental Material

Neurophysiological evidence for rapid processing of verbal and gestural information in understanding communicative actions

Rosario Tomasello[1,2,3]*, Cora Kim[1], Felix R. Dreyer[1], Luigi Grisoni[1] and Friedemann Pulvermüller[1,2,3,4]

[1] Brain Language Laboratory, Department of Philosophy and Humanities, WE4
Freie Universität Berlin
Habelschwerdter Allee 45
14195 Berlin
Germany

[2] Berlin School of Mind and Brain, Humboldt Universität zu Berlin
Luisenstraße 56
10117 Berlin,
Germany

[3] Cluster of Excellence 'Matters of Activity. Image Space Material', Humboldt Universität zu Berlin
Unter den Linden 6
10099 Berlin,
Germany

[4] Einstein Center for Neurosciences
Charitéplatz 1
10117 Berlin
Germany

*Corresponding author. Address for correspondence:

Brain Language Laboratory
Department of Philosophy and Humanities, WE4
Freie Universität Berlin
Habelschwerdter Allee 45
14195 Berlin, Germany
Tel.: +49 (0) 30 838 51984
Tomasello.R@fu-berlin.de
ORCID: 0000-0001-8414-2644

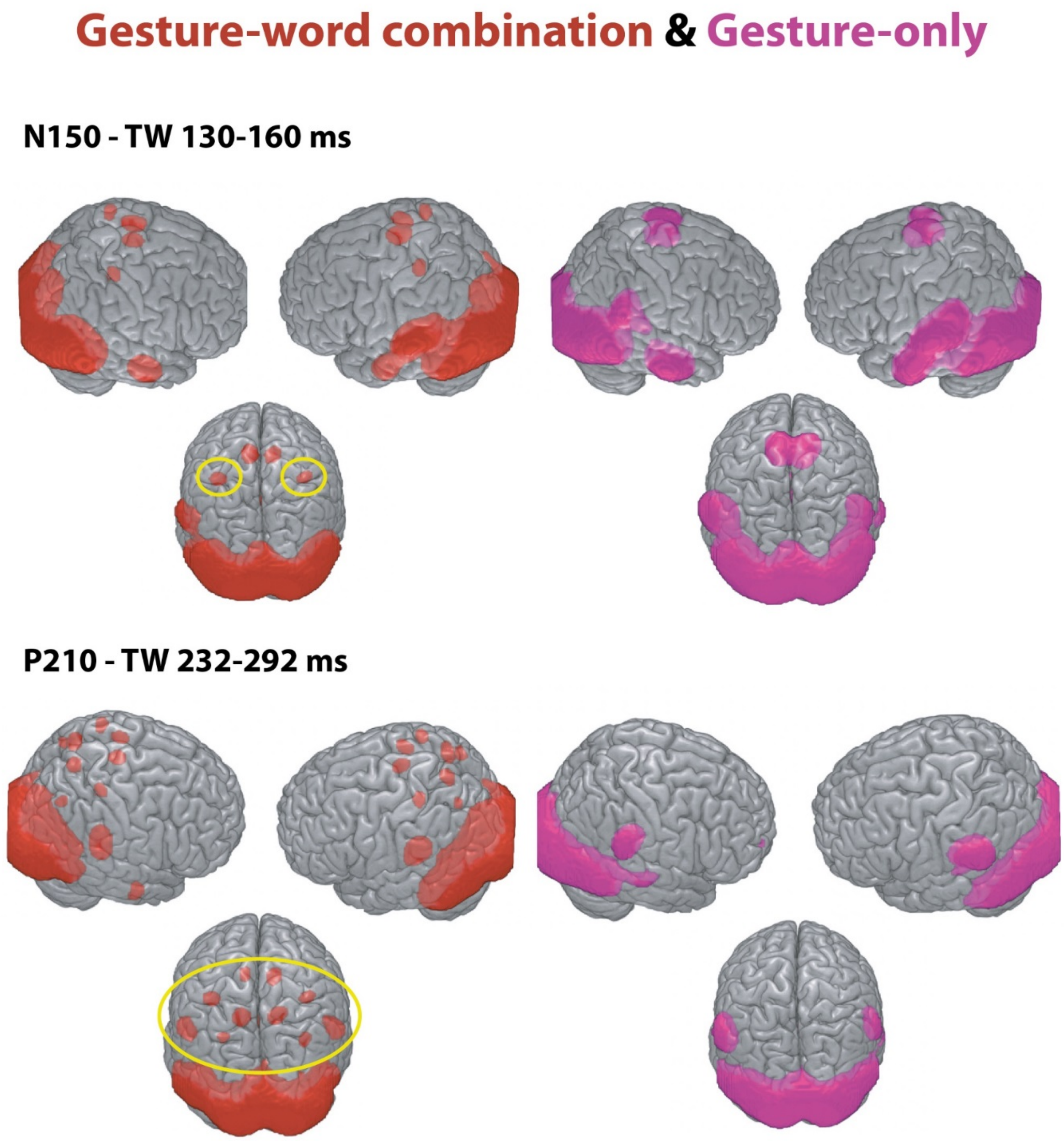

Figure S1: Cortical of activation of pointing and give-me gestures collapsed together for gesture-word composition (red) and gesture-only (violet) conditions at two different latencies, the N150 and P210 time windows. All colored clusters were significantly active (*t*-tests, $p < 0.001$, uncorrected). Round yellow circles highlight the additional cortical areas active for gesture-word composition compared to gesture alone.
